# Construction and characterization of a cardiac organoid ARVC disease model with its application in gene therapy targeting *PKP2*

**DOI:** 10.1016/j.gendis.2026.102184

**Published:** 2026-04-10

**Authors:** Yanan Zhu, Hui Zhang, Zhen Qi, Xinyue Ding, Sijie Yao, Xuan Zhao, Junqing Gao, Lina Xing, Xin Wang, Zongjun Liu

**Affiliations:** a Department of Cardiology, Putuo Hospital, Shanghai University of Traditional Chinese Medicine, Shanghai 200062, China; b Shanghai Key Laboratory of Regulatory Biology, School of Life Sciences, East China Normal University, Shanghai 200241, China; c Department of GCP Office, Shanghai Ninth People's Hospital, Shanghai Jiao Tong University School of Medicine, Shanghai 200011, China; d ACROBiosystems Inc, Beijing 100176, China

Arrhythmogenic right ventricular cardiomyopathy (ARVC) is an inherited genetic cardiomyopathy characterized by lethal arrhythmias and sudden cardiac death with an estimated incidence of 1:2000 to 1:5000 and a male predominance.^1^ As an autosomal inherited disease with familial susceptibility, pathogenic mutations in desmosomal genes, particularly *PKP2* (encoding plakophilin-2), have become the primary disease drivers.^2^ Clinical management of ARVC remains challenging due to the phenotypic heterogeneity and limited patient cohort sizes, with currently no approved therapies targeting its genetic etiology.^3^ Consequently, novel approaches and more human-like *in vitro* models targeting ARVC-associated genes are urgently needed. Recent advances in three-dimensional (3D) organoid technology have enabled the reconstruction of human tissue-like architectures and microenvironments, significantly enhancing disease modeling capabilities.^4^ Furthermore, human induced pluripotent stem cells (hiPSCs) derived from patients with unique genetic backgrounds provide unparalleled opportunities to elucidate disease mechanisms, particularly in cardiovascular research.^5^ Therefore, developing human cardiac organoids with specific pathogenic mutations holds significant scientific and translational value.

Additional results and methods are included in the Supplementary data. This study identified a novel heterozygous truncating mutation (c.983dupG) in *PKP2* among ARVC patients, resulting in a frameshift variant (Fig. S1). Peripheral blood mononuclear cells (PBMCs) were isolated from the venous blood of the ARVC patient (*PKP2*^*c.983dupG/WT*^, *PKP2*^*+/−*^) and his father (WT), followed by reprogramming into hiPSCs (Fig. 1A). After establishing these two hiPSC lines, comprehensive characterization was performed through karyotype analysis, Sanger sequencing, teratoma formation assays, and immunofluorescence staining for pluripotency markers (Fig. S2). These results confirmed the successful reprogramming of PBMCs from both the healthy donor and ARVC patient, demonstrating genomic stability, correct mutation status, and pluripotent differentiation potential.

**Figure 1:**
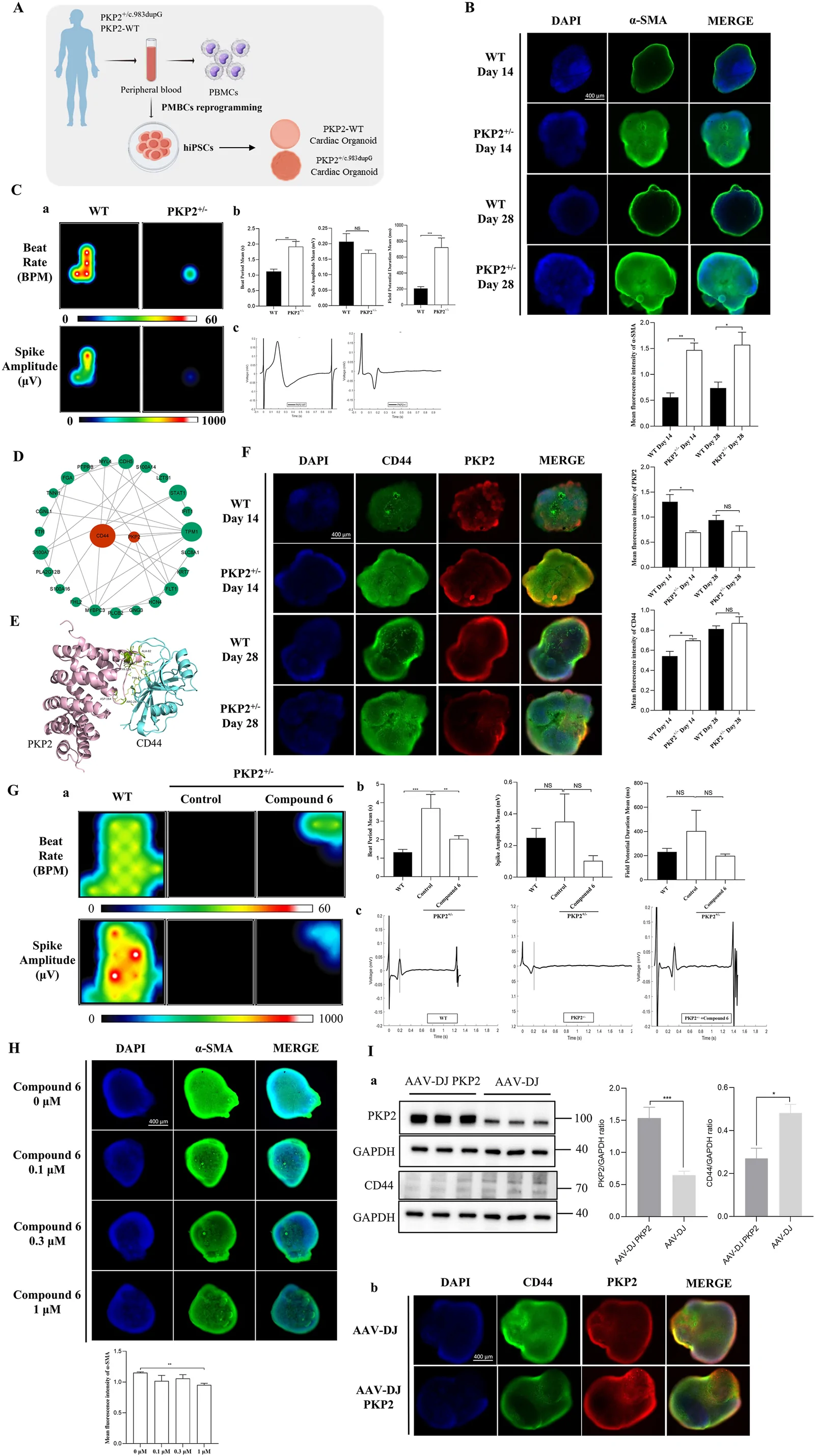
Human ARVC organoids reveal CD44 as the core pathogenic mediator in PKP2-deficient cardiomyopathy. **(A)** Scheme of human induced pluripotent stem cells (hiPSCs)-derived cardiac organoids generation from peripheral blood mononuclear cells (PBMCs). **(B)** Immunofluorescence staining for the myofibroblast cell marker α-SMA in cardiac organoids from the WT or PKP2^+/−^ group at various developmental stages (Day 14 and Day 28). **(C)** Comparable electrophysiology in WT (black) and PKP2^+/−^ (white) cardiac organoids by using MEA. Typical heatmap of the excitatory firing rate and spike amplitude (a), assessment of beat period, spike amplitude, and field potential duration (b), and visualization of field potential (c). **(D)** The protein–protein interaction (PPI) analysis of genes with significant differences. **(E)** The prediction of PPI between CD44 and PKP2. **(F)** Immunofluorescence results of cardiac organoids. **(G)** The MEA analysis of cardiac organoids with the CD44 inhibitor compound 6 (antitumor agent-109). Typical heatmap of the excitatory firing rate and spike amplitude (a), assessment of beat period, spike amplitude, and field potential duration (b), visualization of field potential (c). **(H)** Immunofluorescence results of CD44 inhibition in PKP2^+/−^ cardiac organoids. **(I)** Protein expression of PKP2 restoration in PKP2^+/−^ cardiac organoids. Western blot analysis of PKP2 and CD44 in PKP2-restored cardiac organoids. GAPDH served as a loading control (a). The results of overall immunofluorescence of PKP2 and CD44 in PKP2^+/−^ cardiac organoids with AAV-DJ or AAV-DJ PKP2 (b). The results are expressed as the percentage of means ± SEM. (*n* ≥ 3). *∗p* < 0.05*, ∗∗p* < 0.01*,* and *∗∗∗p* < 0.001. Scale bar: 400 μm.

Cardiac organoids were subsequently generated from hiPSCs with either *PKP2*^*+/−*^ or WT genetic backgrounds to model ARVC pathogenesis. Notably, during differentiation, *PKP2*^*+/−*^ organoids exhibited progressive structural abnormalities from Day 5, including irregular morphology and disintegration of intercellular connections within myocardial tissue. This phenotype aligns with the known role of PKP2 in maintaining intercalated disc integrity. Concomitantly, *PKP2*^*+/−*^ organoids showed a significantly reduced expression of desmosomal proteins (PKP2, DSG2, DSC2, and JUP) and markedly elevated levels of α-SMA, the fibrosis biomarker, compared to WT controls (Fig. 1B; Fig. S3). This disease-relevant phenotype cluster, characterized by structural disintegration, desmosome deficiency, and spontaneous fibrogenesis, faithfully recapitulates the progressive myocardial fibrotic remodeling observed in ARVC patients.

Except for myocardial fibrosis, ventricular arrhythmia is also the hallmark of ARVC. Therefore, to evaluate whether this *PKP2*^*+/−*^ cardiac organoid model could recapitulate this key clinical phenotype, we performed functional electrophysiological profiling using multi-electrode arrays (MEAs). The results showed that compared with the WT group, the field potentials of the *PKP2*^*+/−*^ cardiac organoids exhibited wider QRS tachycardia, with a relatively lower rate (Fig. 1C). This electrophysiological profile mirrors the ventricular tachycardias with broad QRS complexes observed in ARVC patients. While high-density microelectrode arrays (HD-MEAs) could offer superior spatial resolution, their use was precluded under the current experimental setup due to technical constraints. Collectively, our model recapitulates both the structural pathology and functional electrophysiological deficits characteristic of human ARVC.

To explore the underlying molecular mechanism of PKP2 in ARVC, a joint analysis of transcriptomic and proteomic profiling of cardiac organoids was conducted. Bulk multi-omics analysis revealed significant alterations in lipid metabolism, the immune system, and intercellular connections-related pathways. MAPK, PI3K/AKT, and PPARγ pathways were enriched in the KEGG analysis on Day 14. Notably, these pathway disruptions were temporally restricted: while profoundly altered on Day 14 (coinciding with early structural abnormalities), they returned to baseline by Day 28 (Fig. S4). This biphasic pattern suggests that the early pathway dysregulation directly contributes to the initial pathogenesis, and the late-phase normalization may represent compensatory feedback mechanisms masking ongoing pathology. Taken together, these results highlight the therapeutic relevance of early intervention in ARVC.

To investigate protein–protein interactions (PPIs) in ARVC pathogenesis, we integrated 43 differentially expressed genes (DEGs) from Day 14 and 9 DEGs from Day 28 for PPI network construction (Fig. 1D). After eliminating disconnected nodes, the PPI network retained a total of 25 nodes and 66 edges. CD44 was identified as the hub gene of the PPI network through the betweenness value calculation. To validate this finding, we predicted the CD44-PKP2 interaction using AlphaFold3, which revealed a high-affinity binding interface stabilized by five hydrogen bonds (Table S1; Fig. 1E; CD45: blue, PKP2: red). Crucially, *PKP2* mutation triggered significant upregulation of CD44 expression, a previously unreported pathogenic mechanism (Fig. 1F). Mechanistically, pharmacological inhibition of CD44 with compound 6 restored cardiac electrophysiological function and attenuated myocardial fibrosis in *PKP2*^*+/−*^ organoids with no effect on the cell viability (Fig. 1G and H; Fig. S5). Collectively, these results establish CD44 as a central mediator of PKP2-associated ARVC and demonstrate the therapeutic potential of CD44 inhibition.

Furthermore, this study demonstrates the therapeutic potential of *PKP2* gene restoration in the human cardiac organoid model of ARVC. AAV-DJ-mediated PKP2 restoration effectively rescued PKP2 expression in ARVC cardiac organoids. Significantly, *PKP2* restoration downregulated CD44 expression (Fig. 1I; Fig. S6), consistent with CD44's established role as a pathogenic driver. Collectively, these findings indicate that CD44 suppression constitutes a key mechanistic component of AAV-mediated *PKP2* therapy in ARVC.

In conclusion, this study innovatively constructed patient-derived *PKP2*-mutant cardiac organoids as a physiologically relevant 3D model for ARVC pathogenesis research. Using this model, we identified CD44 as a central pathogenic mediator in ARVC with *PKP2* mutations. Moreover, we carried out AAV-DJ gene therapy targeting *PKP2* based on the above organoid model, which showed beneficial effects and revealed the potential of repairing *PKP2* mutations to treat ARVC patients. Critically, AAV-DJ-mediated *PKP2* gene therapy not only rescued PKP2 expression but also normalized CD44 overexpression, demonstrating a dual-pathway therapeutic mechanism. These findings position CD44-targeted interventions and *PKP2* gene correction as translational strategies for the precision treatment of ARVC.

## CRediT authorship contribution statement

**Yanan Zhu:** Writing – original draft, Visualization, Validation, Supervision, Software, Resources, Project administration, Methodology, Investigation, Funding acquisition, Formal analysis, Data curation, Conceptualization. **Hui Zhang:** Writing – original draft, Visualization, Validation, Software, Resources, Project administration, Methodology, Formal analysis, Data curation. **Zhen Qi:** Project administration, Methodology, Investigation. **Xinyue Ding:** Investigation. **Sijie Yao:** Methodology, Investigation. **Xuan Zhao:** Methodology, Investigation. **Junqing Gao:** Investigation. **Lina Xing:** Supervision, Funding acquisition. **Xin Wang:** Writing – review & editing, Supervision. **Zongjun Liu:** Writing – review & editing, Supervision, Funding acquisition, Conceptualization.

## Ethics declaration

All experimental protocols on patients were approved by the Ethics Committee of Putuo Hospital, Shanghai University of Traditional Chinese Medicine (reference number: PTEC-A-2022-13 (S) −1), and were conducted in accordance with the ethical standards of the Declaration of Helsinki.

## Funding

This work was supported by the Natural Science Foundation of Shanghai (China) (No. 23ZR1456500) and the 10.13039/501100012566Health System Independent Innovation Science Foundation of Shanghai Putuo District (China) (No. ptkwws202101, 2023ysxk01).

## Conflict of interests

The authors declare the following financial interests/personal relationships which may be considered as potential competing interests: Zhen Qi is currently employed by ACROBiosystems Inc, Beijing. The remaing authors declare no conflict of interests.
