# Supplementary material for: Construction and characterization of a cardiac organoid ARVC disease model with its application in gene therapy targeting PKP2
Source: Genes Dis. 2026 Apr 10;14(1):102184. doi: 10.1016/j.gendis.2026.102184 (PMC13571223; doi:10.1016/j.gendis.2026.102184)
Supplement: Supplementary file 1 [file mmc1.docx]

**Materials and methods**

***Reagents***

Rho kinase inhibitor Y-27632 was bought from Beyotime (Shanghai, China). BMP4, CHIR-99021, LY294002, activin A, FGF basic, IWR-1, retinoic acid, and compound 6 (HA-CD44 interaction inhibitor 1, HY-149898) were supplied by MCE (State of New Jersey, USA). GlutaMAX™ (100X) and RPMI 1640 medium were purchased from Thermo Fisher Scientific (Waltham, MA, USA). mTeSR Plus and Gentle Cell Dissociation Reagent were bought from STEMCELL (Vancouver, Canada). Bovine serum albumin was bought from Bovostar. L-ascorbic acid 2-phosphate was bought from GLPBIO (Montclair, CA, USA). Sodium pyruvate was bought from Solarbio (Beijing, China). Insulin was bought from Pricella (Wuhan, China). The U-shaped ultralow-attachment 96-well plate (7007) and Matrigel (354277) were obtained from Corning Inc. (Bedford, MA, USA). Phospho-p44/42 MAPK (ERK1/2) (cat#4370), p44/42 MAPK (ERK1/2) (cat#4695), COX IV (cat#4844), γ-Catenin Antibody/JUP (cat#2309S) were purchased from Cell Signaling Technology (Danvers, Massachusetts, U.S.). Anti-cardiac troponin T (ab209813), Anti-CD31 (ab9498), Anti-alpha smooth muscle actin (ab124964), anti-AKT1 (phospho S473) (ab81283), anti-desmoglein 2/DSG2 (ab150372), anti-desmocollin 2/DSC2 (ab95967), anti-plakophilin 2 / PKP2 (ab223757), anti-desmin (ab32362), anti-GAPDH (ab181602), and anti-CD44 (ab51037) were purchased from Abcam (Cambridge, UK). HRP-labeled goat anti-rabbit secondary antibody (H+L) (A0208) was purchased from Beyotime (Shanghai, China). PPARγ (A11183) was obtained from ABclonal (Shanghai, China).

***Clinical evaluation***

The ECG, echocardiography, and family history of ARVC patients were recorded. After obtaining the signed informed consent form, 5 mL of peripheral blood was used for DNA extraction according to standard methods. Genetic testing was performed as previously described [6]. All experimental protocols on patients were approved by the Ethics Committee of Putuo Hospital, Shanghai University of Traditional Chinese Medicine (reference number: PTEC-A-2022-13 (S) -1), and were conducted in accordance with the ethical standards of the Declaration of Helsinki.

***Cell culture and reprogramming***

PBMCs were collected from the venous blood of one ARVC patient and his father. Both subjects had given informed consent to be included in the study prior to their participation. PBMCs were collected from the patient’s venous blood by performing standard density gradient centrifugation at 400 g for 30 mins using Ficoll Hypaque (G&E Healthcare). Cells were cultured in PBMCeasy^®^ blood cell activation medium (Cellapy, Cat. CA3408025) for 4 ~ 6 days. Human iPSCs were generated by reprogramming the PBMCs using the Reproeasy hiPSC reprogramming kit (Cellapy, Cat. CA5002002). Three days later, the medium was half-changed with PBMCeasy^®^ blood cell activation medium. After one week of reprogramming, iPSC colonies were formed, and the medium was replaced with a Reproeasy reprogramming medium (Cellapy, Cat. CA5003050). Six days later, iPSC colonies were manually isolated and expanded in PGM1 human pluripotent stem cell culture medium (Cellapy, Cat. CA1007500) on plates pre-coated with Matrigel at 37 °C and 5% CO_2_. The medium was daily renewed, and cells were passaged with Gentle Cell Dissociation Reagent.

The PBMC-derived hiPSCs were grown in hESC-Qualified Matrix and Matrigel-coated wells. The human hiPS received fresh mTeSR Medium daily and were passaged at 80–95% confluency levels. Briefly, the medium was aspirated and passage of the cells was performed with Gentle Cell Dissociation Reagent for 5 mins at 37 °C with 5% CO_2_ in a humidified atmosphere. After incubation, 3 mL of mTeSR medium supplemented with 10 μM Rho kinase inhibitor (ROCK I) Y-27632 was added to the dissociated cells and transferred to a 15 mL tube. Cells were centrifuged for 5 mins at 300 g. Finally, the cells were seeded in mTeSR medium supplemented with 10 μM ROCK I. The medium was replaced on the next day with plain mTeSR.

***Karyotype analysis***

Karyotype analysis for hiPSCs was performed using G-banding. According to the International System for Human Cytogenetic Nomenclature (ISCN, 2016), approximately 20 metaphase chromosome spreads and G-banding tests were performed on humans using Video TesT-Karyo 3.1.

***Immunofluorescence***

The hiPSCs were grown on Matrigel-coated coverslips in 24-well culture plates and fixed with 4 % (w/v) paraformaldehyde (PFA) at room temperature for 15 mins. The hiPSCs were permeabilized in 0.5 % Triton X-100 for 20 mins and blocked in 3% BSA for 30 mins. Then the hiPSCs were incubated primary antibodies: Oct4, Nanog, Sox2 (Santa Cruz, USA) in 3 % BSA overnight at 4 °C. After washing three times with PBS, the secondary antibodies Alexa Fluor® 594 Goat Anti-Mouse IgG (H+L) or Alexa Fluor® 594 Goat Anti-Rabbit IgG (H+L) (Santa Cruz, USA) were added and incubated for 1 h. Nuclei were stained with DAPI (VECTOR, USA) for 10 mins at room temperature. Images were obtained using the fluorescence inverted microscope (Axio Vert.A1, Zeiss) shown in Fig. 2, with a scale bar of 200 μm.

Human cardiac organoids were fixed with 4% paraformaldehyde (PFA) at 4 ℃ for 12 h. Then, the organoids were permeabilized in 0.5% Triton X-100 for 30 mins and blocked in 1% BSA for 4 h. The organoids were incubated with the primary antibody overnight at 4 ℃ and washed three times with DPBS. The secondary antibody was added to the tube and incubated at room temperature for 2 h under dark conditions. After being washed three times with DPBS, the samples were then visualized under a fluorescence microscope (Revolve Generation 2, ECHO). The antibodies involved in this experiment included rabbit anti-cTnT (Abcam, ab209813), mouse anti-CD31 (Santa Cruz Biotechnology, sc-376764), mouse anti–plakophilin 2 (Santa Cruz Biotechnology, sc-393711), rabbit anti-alpha smooth muscle (Abcam, ab5694), and rabbit anti-CD44 (Abcam, ab51037).

***Mutation analysis***

The genomic DNA of hiPSC was isolated as described above. Bidirectional Sanger sequencing analysis was conducted with PCR products. The heterozygous mutations in PKP2 were validated using Sanger sequencing.

***Teratoma formation assay***

All experimental protocols on NOD-SCID mice were approved by the Beijing Viewsolid Biotechnology Co., Ltd. (reference number: VS2126A01206). HiPSCs at 10 passages were passaged with the Gentle Cell Dissociation Reagent. Approximately 1 × 10^7^ cells were suspended in PBS and injected into the 4-week-old male non-obese diabetic/severe combined immunodeficiency (NOD-SCID) mice into the left dorsal flank. Mice's health and tumor growth were controlled every day. Two to three months after the injection, teratomas were excised. Hematoxylin-eosin (HE) staining was performed as described previously [7].

***Organoid culture***

According to previous reports, heart-forming organoids (HFOs) can be derived from human pluripotent stem cells (hPSCs) [8]. Some optimizations on the differentiation condition were also conducted. The differentiation protocol started when human iPSCs reached 80 - 95% confluency. Cells were detached from Matrigel-coated wells using Gentle Cell Dissociation Reagent and were seeded in a U-shaped ultralow-attachment 96-well plate in mTeSR medium with 10 μM ROCK I at a density of 7500 to 9000 cells per well. The plate was centrifuged for 5 mins at 300 g. After 24 h incubation at 37 °C and 5% CO_2_ in a humidified atmosphere, an embryoid body (EB) was formed. The EBs with a diameter between 350 nm and 500 nm were selected for the next step. Then the medium was replaced with differentiation medium A, which contains LY294002, BMP-4, CHIR-99021, Activin A, and insulin. Two days later, the differentiation medium B containing BMP4, IWR1, Retinoic acid, FGF2, and insulin was replaced daily, lasting for 4 days. Then, the EBs were cultivated in differentiation medium C containing FGF2 and insulin, and the medium was replaced daily for 5 days. Lastly, the EBs were cultured in a maintenance medium with insulin, and the medium was replaced daily. The differentiation of cardiac organoids is shown in Fig. 3A.

***Western blot***

The iPSC-derived cardiac organoids were collected in a 1.5 mL tube and then lysed in a Lysis buffer containing phosphatase inhibitor, protease inhibitor, and PMSF. For immunoblotting, 10 μg of protein extract per well was loaded onto Tanon™ 4 – 12% Bis-tris Precast Gel and then transferred to PVDF membranes. The membranes were blocked in 5% non-fat milk in Tris-buffered saline with 0.1% Tween-20 at room temperature for 1 h and incubated overnight at 4 ℃ with primary antibodies. After 3 times washing with TBS-T, the membranes were incubated with secondary antibodies at room temperature for 1 h. The membranes were then washed three times with TBS-T. HRP-labeled secondary antibodies were used in combination with the Clarity Western ECL Substrate kit for visualization. Immunoblots were imaged using Tanon-4600, and all blots were analyzed using the ImageJ software (NIH, Bethesda, MD).

***MEA assay***

The MEA assay was used to evaluate the effects of the PKP2 c.983dupG heterozygous mutation on the electrophysiological properties in human cardiac organoids. Briefly, the cardiac organoids were cultured in CytoView MEA 24 plates (Axion BioSystems, USA) pre-coated with Matrigel, with one organoid seeded per well for potential recording. The MEA device automatically adjusted and controlled the environment (37 °C and 5% CO_2_) to maintain the temperature and medium pH. The data were obtained by quantifying the beat rate, field potential duration, and spike amplitude from MEA recordings using the Maestro Pro multi-well MEA platform and cardiac analysis tool (Axion BioSystems, USA). These parameters correspond to the organoids' beating frequency and signal intensity, respectively.

***Bulk human cardiac organoids RNA-seq and data analysis***

Ten human cardiac organoids from the PKP2 c.983dupG heterozygous mutation and the WT group were randomly selected and pooled together as one mixed sample. One mixed sample in each group was used for total RNA extraction using the TRIzol® Reagent according to the manufacturer’s instructions (Magen). RNA quality was evaluated by an Agilent Bioanalyzer 4150 system (Agilent Technologies, CA, USA). The library preparations were sequenced on an Illumina Novaseq 6000 (or MGISEQ-T7), and 150 bp paired-end reads were generated. The data generated from the Illumina (or BGI) platform were used for bioinformatics analysis. Raw data were filtered to get clean reads using FastQC (v0.20.0). Then, clean reads were separately aligned to the reference genome with orientation mode using HISAT2 software (http://daehwankimlab.github.io/hisat2/) to obtain mapped reads. FeatureCounts (http://subread.sourceforge.net/) were used to count the number of reads mapped to each gene. Then FPKM of each gene was calculated based on the length of the gene and the read count mapped to this gene. Differential gene expression analysis between the PKP2 c.983dupG heterozygous mutation and the WT group was performed using the DESeq2 package (v1.34.0). *P* values were adjusted using the Benjamini–Hochberg method. DEGs was identified with |log2FC| ≥ 1 and *P*-value < 0.05. To annotate the functions of DEGs, the GO function and KEGG pathway enrichment analysis were performed using the OmicStudio tools at the website https://www.omicstudio.cn/tool.

***Bulk human cardiac organoids proteomics analysis***

For proteomics analysis, ten cardiac organoids from the PKP2 c.983dupG heterozygous mutation and the WT group were randomly selected and pooled together as one mixed sample. In short, the samples were first homogenized by MP FastPrep-24 homogenizer (24 × 2,6.0M/S, 60 s, twice), and then SDT buffer (4 % SDS, 100 mM Tris-HCl, pH 7.6) was added. The lysate was centrifuged (14,000 g, 4 °C, 40 mins) to get the supernatant. Then the supernatant was quantified with the BCA protein quantitative kit. DTT (161‐0404, Bio‐Rad) and Iodoacetamide (IAA, 163‐2109, Bio‐Rad) were used to break the disulfide bond and reductively alkylate the protein. Next, the samples were transferred to the filters (Microcon units, 10 kDa) and were washed three times with 100 μL UA buffer and then washed twice with 100 μL of NH_4_HCO_3_ buffer (25 mM). Finally, trypsin was added to the samples, and the resulting peptides were collected as a filtrate. The peptides were desalted on C18 Cartridges (Empore™ SPE Cartridges MCX, 30UM, Waters). For DIA experiments, iRT (indexed retention time) calibration peptides were spiked into the sample. The peptides from each sample were analyzed by Orbitrap^TM^ Astral^TM^ mass spectrometer (Thermo Scientific) connected to a Vanquish Neo system liquid chromatography (Thermo Scientific) in the data-independent acquisition (DIA) mode.

DIA data was analyzed with DIA-NN 1.8.1. The protein sequences of the selected differentially expressed proteins were locally searched using the NCBI BLAST+ client software (ncbi-blast-2.2.28+-win32.exe) and InterProScan to find homologous sequences. The criteria for screening differential expression genes (DEPs) were that fold change was greater than 1.2 (up-regulated proteins) or < 0.83 (down-regulated proteins) and *P*-value < 0.05. GO function and KEGG pathway enrichment analysis were performed using the OmicStudio tools at the website https://www.omicstudio.cn/tool.

***Protein-protein interaction analysis***

The protein-protein interaction (PPI) information of the studied proteins was retrieved from STRING software (http://string-db.org/). The results were downloaded in the XGMML format and imported into Cytoscape software (http://www.cytoscape.org/, version 3.2.1) to visualize and further analyze functional PPI networks. Furthermore, the betweenness of each protein was calculated to evaluate the importance of the protein in the PPI network.

***Prediction of protein-protein interaction***

The 3D structure (resolution < 3A) and FASTA sequence were obtained through the PDB database (https://www.rcsb.org/). AlphaFold3 was used to predict the interaction between the target protein (PKP2) and the candidate protein. The template modeling score was measured using the predicted template modeling (pTM) score and the Interface Predicted Template Modeling (ipTM) score. pTM+ipTM > 0.75 indicates the presence of interactions between these proteins. Pymol software was used to map the interactions between the protein complexes.

***AAV restoration***

AAV vectors (serotype DJ) encoding human PKP2 or empty vectors were generated in collaboration with Genomeditech (Shanghai, China). The viral loads (v.l.) of 1E10 viral genomes (v.g.) per organoid were delivered with an AAV-DJ vector expressing the WT human PKP2 gene under the control of a cytomegalovirus (CMV) immediate-early promoter (AAV-DJ PKP2). After AAV transduction, the medium was changed to a regular maintenance medium.

***Statistical analysis***

Statistical analyses were performed using the GraphPad Prism 9.0 software (GraphPad, Avenida, CA, United States). Results were expressed as the means ± S.E.M. (standard error of measurement). Normality and lognormality tests were performed first to determine whether a dataset was normally distributed. Ordinary one-way ANOVA followed by the Dunnett post hoc test and Student’s t-test were performed to analyze the data for the biochemical test. *P* < 0.05 was considered statistically significant.

**Supplementary table legends**

**Table S1** The prediction of protein-protein interaction between CD44 and PKP2.

| **Hydrogen bond** | |
| --- | --- |
| PKP2 (Red) | CD44 (Blue) |
| ASP-164 | ARG-24 |
| TYR-156 | TYR-25 |
| ASP-155 | TYR-62 |
| ASP155 | ALA-82 |
| ASP155 | ILE-79 |

**Supplementary Figure legends**

**Supplementary Figure 1** Identification of PKP2 mutation. A, ARVC’s family diagram (box: male, circle: female). The variant is in solid black. Ⅰ, Ⅱ, and Ⅲ represent different generations. B, Sanger sequences surrounding the mutation (red arrow) within the PKP2 gene. C, Patient’s electrocardiogram, Premature ventricular triad (a) and Paroxysmal ventricular tachycardia (b). D, The results of cardiac echocardiography.

**Supplementary Figure 2** Characterization of PBMC reprogramming hiPSC pluripotency. A, Scheme of human induced pluripotent stem cells (hiPSCs) generation from peripheral blood mononuclear cells (PBMCs). B, Representative images of iPSC generation. PBMCs from the patient (a). Small colonies after transducing on day 7 (b). The colonies after picking (c). Scale bar, 400 µm. C, Karyotype analysis for WT, and PKP2^+/-^ iPSCs. D, Sanger sequence of WT, and PKP2^+/-^ iPSCs. E, HE staining of the teratoma formation assay. F, Immunofluorescence staining of multipotent genes NANOG, OCT4, and SOX2 in WT and PKP2^+/-^ iPSCs.

**Supplementary Figure 3** Characterization of hiPSCs-derived human cardiac organoids. A, Morphological pictures of hiPSCs-derived cardiac organoids on different days by using a microscope (n = 10 ~ 30). B, Western blot analysis of PKP2^+/-^ and WT cardiac organoids at various development stages (Day 14 and Day 28). Above each panel are representative immunoblots and PKP2, DSG2, DSC2, and JUP quantifications. GAPDH served as a loading control. The results are expressed as the percentage of means ± S.E.M. (n = 3 ~ 6). ^*^*P* < 0.05, ^**^*P* < 0.01, and ^***^*P* < 0.001.

**Supplementary Figure 4** Integration of transcriptomic and proteomic analyses at different stages of PKP2^+/-^ and WT cardiac organoids development. A, Comparison of human cardiac organoids-related mRNA and protein changes on Day 14 (a) and 28 (b), *P＜0.05*. These can be divided into four groups depending on the direction of change, with decreased RNA and increased protein (part 1), decreased RNA and decreased protein (part 7), increased RNA and increased protein (part 3), and increased RNA and decreased protein (part 9). B, The KEGG and GO analysis of PKP2^+/-^ and WT cardiac organoids on Day 14 (a and b). C, Western blot analysis of PKP2^+/-^ and WT cardiac organoids on Day 14 and Day 28. Above each panel are representative immunoblots and AKT, MAPK, and PPARγ quantifications. GAPDH served as a loading control. The results are expressed as the percentage of means ± S.E.M. (n = 3 ~ 6). *^*^P* < 0.05, and *^**^P* < 0.01.

**Supplementary Figure 5** The CCK-8 result of the CD44 inhibitor Compound 6 at different dosages on PKP2^+/-^ cardiac organoids. The results are expressed as the percentage of means ± S.E.M. (n = 5).

**Supplementary Figure 6** AAV-mediated PKP2 restoration in PKP2^+/-^ cardiac organoids. A, Different doses of AAV-DJ carrier at different times (n = 3). B, The structure of the AAV-DJ PKP2 plasmid. C, Flowchart of AAV-mediated PKP2 restoration in PKP2^+/-^ cardiac organoids.
